# Kate Mountstephen

**Published:** 1887-09-10

**Authors:** C. G. Furley


### KATE MOUNTSTEPHEN.

By C. G. Furley.

(Continued from p. 386, vol. ii.)

### Chapter III.—A Hard Question.

"I never met any one quite like you, Miss Mountstephen."

"That's a very dubious compliment, Mr. Barnett; in fact, it is a remark that has often been made about me, and usually with unflattering intention. To be 'not quite like other people' is to be eccentric, and eccentricity is a misfortune, when it isn't a crime."

"A misfortune to be superior to other women, or a crime to be helpful where they are helpless!" exclaimed Harry, indignantly.

"But I'm not superior: I admit that myself; I am only different," Kate explained. "I took up nursing merely because I liked it, or at least because I could not bear to see the poor creatures around me die, simply because those who had charge of them had not knowledge and common-sense enough to carry out my father's orders. And I picked up the little anatomy and surgery I know in a desultory manner, because it interested me and helped me in my work. It is no more creditable to me to be able to do something in that way than it is for other girls to play Chopin or paint on china."

"It's rather more useful, though."

"I don't know. Art is a valuable adjunct of life."

"Health and freedom from pain are more valuable still—at least, from my point of view," said Harry.

"Ah! but your point of view may be considered a very narrow, indeed a personal one."

"Perhaps it is; but, after all you did for me, how can I think anything but that you are the noblest, and sweetest, and dearest girl in the world? And—and—oh! Kate, you know what I mean," concluded the young man, irrelevantly.

Nevertheless Kate did know what he meant; for what his stammering words left unuttered his glistening eyes said as plainly as need be.

Harry Barnett was quite well now. Three weeks had passed since he had met with the accident, and a week ago he had been well enough to leave Coteswold to attend his uncle's funeral; for old Mr. Barnett had never recovered from the shock of his baby son's death, and after lingering for more than a week on the shore of the dark river, he had crossed over to join his child in the further world. His death left Harry again, and this time inalienably, a rich man. But as yet the change in his fortunes had impressed him very little. Grief for the sad circumstances of his uncle's death had occupied part of his thoughts; a new and yet uncertain joy had filled the rest.

That Harry should have fallen in love with Kate Mountstephen was not a very remarkable fact. "How on earth could he help it?" as poor Captain Mitchell said irritably, when his friend confided his passion to him, in a tone that suggested the idea that he had made the most wonderful discovery and performed the most wonderful feat that mortal man had ever had power and fortune to do.

"You needn't seem so surprised at it, Barnett," the captain had said; "I knew from the moment she set your arm that this would be the end of it. And it's no particular credit to you either; other people have fallen in love with Miss Mountstephen before you, and will do it after you, I have no doubt. And, under the circumstances, how on earth could you help it?"

Perhaps the captain's bad temper sprang from the fact, perceived by the rejected lover before it was guessed at by Harry, or even suspected by Kate herself, that she would not be indifferent to Barnett's love.

"Why on earth that should be, I don't know," he grumbled to himself. "If I had thought that was the way to win her, I would have broken my arms and legs thrice over last winter. But hang it all, she doesn't fall in love with all those village children whom she pulls through their fevers and whooping-coughs!"

But as the captain might have known, a good-looking young man is not quite the same as a peasant child, even to the most exceptional of women.

The days of his convalescence were, as Harry often said to himself in the years that immediately followed, the happiest he had ever known or was ever likely to know. It was then that he learned the depths of Kate Mountstephen's character; the strong and tender feeling that lay beneath the occasional abruptness of her frank and careless manner; the serious thought that dwelt behind her ready speech. Not that he analysed her character much; the love-mist was in his eyes, and he was content to see in her the woman he loved and wanted for his wife.

Though he knew his uncle was ill, the news of his death had come upon him with a shock, so full had his mind been of happier subjects. He went to London for the funeral, but returned to Coteswold within a few days—"to thank Dr. Mountstephen for all his kindness," he said. "And to rob me of my most treasured possession by way of gratitude," added the doctor with a touch of bitterness, when, on the evening of his arrival, Harry asked him for his daughter's hand.

"But since you have her consent to the marriage, I suppose mine must follow," said the old gentleman. "I always do what Katie wants; I have got into the habit of it, and can't rebel even when her wish is to leave me. And if she must marry, I would as soon have you, Barnett, as anybody I know; for you're an honest fellow, and I think you love her sincerely."

"Which wasn't at all the way in which to consent to such a proposal," said Mrs. Jeffreys to her brother-in-law afterwards, reminding him of the words he had used. "Don't you know, William, that Mr. Barnett is one of the richest men in London; and that Kate, though she is a very sweet and noble girl" (the sweetness and nobility had been discovered by Mrs. Jeffreys only recently), "is so very unla—unfem—well, uncommon in her ways, that the chances were very much against her ever getting a husband, not to speak of such a husband as this, whom ninety-nine girls out of a hundred would be glad to jump at."

"If he were ten times as good and a hundred times as rich he wouldn't be too good for my Katie," said the doctor obstinately. "But since she likes him, and he likes her, they must take their own way. If it makes her happy I have nothing to say against it."

"I think you can see that it does make her happy," said Aunt Helen; and indeed it would have required very wilful blindness on the doctor's part not to have observed the new brightness that dwelt in his daughter's eyes, and straightened into smiles the curves of her firm yet sensitive mouth. Harry adored her in true knightly fashion. She was his lady, whose every command he must needs obey gladly—even her command in caprice, as Ruskin advises, had Kate Mountstephen been one who cared to test her power over a man by meaningless tyranny. "Whatever she does it must sure be right," was his creed, a creed which he could hold the more easily because he knew that all her wishes were inspired by a clear judgment and a tender soul. If in spite of this complete trust in her wisdom she was able to look upon him as her master, to whom she in turn would yield obedience as perfect, Harry was content to accept the fact as one of the exquisite miracles of love, and to vow to himself that he would strive to prove not wholly unworthy of her confiding faith.

"One is so horribly tempted in this world just to do what suits oneself best, and let the right and wrong of the matter slide," he said to himself, recalling some Stock Exchange experiences, "but no man would be fit to touch her hand, much less to claim her as his wife, who couldn't take her motto for his own,—'*Fais ce que dois, advienne que pourra.*'"

For a week the two were intensely happy—a week that seemed little more than an hour in passing, and almost an eternity to look back upon. Then Harry said he must go to London; there were many things to be done in regard to the arrangement of his uncle's property which demanded his attention.

"Oh, Mr. Barnett, there's something I have been wanting to tell you about for two days past," said Willis, intruding upon him as he was packing his portmanteau. "You remember that coat as you had on when you was thrown from your horse; Miss Kate had to split the sleeve up, and you bade me give it to Peter." Peter was the doctor's groom and man-of-all-work.

"Yes, Willis, I know I did. I hope you aren't going to quarrel with me on account of that, though I know the coat should have been yours by rights in exchange for that gorgeous shawl in which you wrapped me up instead; I don't think the coat would have fitted you, you know."

"Lord, sir, what a nonsense you do talk. It's well for you, you're agoing to get a nice sensible young lady like our Miss Kate for your wife," said Willis, with a smile at the joke. She had all along looked upon Harry as in part her property, and now that he was engaged to her young mistress her manner to him was almost grandmotherly.

"But what I was going to tell you was this," she went on. "Peter put the coat away in his box, and took it out only the other day, meaning to get me or one of the maids to mend it for him; and as he was looking through the pockets he found this in one of them."

And as she spoke, Willis produced from the pocket of her ample apron a letter—the letter he had received on the morning of his accident, and had first neglected, and then forgotten to open—a perfumed letter whose envelope was decorated with a dainty monogram; a letter which he had recognised at the time as coming from Evelyn Ellis.

Harry turned first red and then pale as he took it from the old woman. The flush came from that feeling of shame which touches most men when in the midst of a new love they are reminded of a forgotten old one; the pallor, from an undefined premonition of trouble that might spring from this prettily written note.

"Why do you bring me this now, Willis?" he exclaimed, involuntarily.

"Why, sir, it's yours; don't you want to have it?"

"Oh yes; it's all right. But I have sometimes thought that if you don't get a letter at the right time it's better not to get it at all."

"And I wish with all my heart he had never got it at all," Willis confided to herself many a time afterwards. "It wouldn't have been right, I know, but if I had known what was inside that envelope, I'd have burned the whole thing, that I would, and taken the consequences here and hereafter."

This was how the letter ran:

"Dearest Harry,—At last our long and apparently hopeless probation is over. Mamma has given her consent to our marriage. I suppose that after all she has sufficient motherly feeling to prefer seeing me married, even to you, to seeing me laid in my coffin; and she knows—at least I have given her more than one proof of my determination—that I will never accept anybody else, no matter how rich he may be. So she has given in; and I can be yours whenever you like to take me.

"I hope you won't think I have grown very plain. I fear I am a great deal thinner than when we parted; but I have fretted so much all this dreary time that I have absolutely pined away. We are in London again, in the same house, so come to me as soon as you can, and I will bid you welcome in the same room where I bade you good-bye. I shan't get a very fine trousseau, for we shall be quite, quite poor, shall we not?

"Always, my own darling Harry, your loving

"Evelyn Ellis."

While Harry was reading the note, Willis, who held masculine packing in very little esteem, had been rearranging the contents of the portmanteau in a manner which seemed to her more in keeping with the orthodox fitness of things; but she turned abruptly from her task when she heard a groan, and saw Mr. Barnett sink into a chair and cover his face with his hand.

"Why, what's the matter, sir?" she cried.

"Nothing—that is, something unexpected. I can't explain to you; but will you ask Miss Kate if I can see her alone at once?"

Willis hurried away to deliver the message and to bring bim some brandy, which she considered to be a specific for all the ills that either flesh or spirit is heir to. But Harry rejected the proffered restorative, and went straightway to meet his betrothed in the morning-room.

"Why, Harry, how formal you have grown!" Kate exclaimed, laughing. "Sending a message to ask if I

can receive you. That is making me more of a queen than I care to be." Then she saw the expression of his face, which seemed suddenly, within a few minutes, to have grown haggard and old, and her tone changed. "Dearest," she exclaimed, "what is the matter? Are you ill? Has anything happened?"

"Yes, something has happened, something I never thought of. Oh, Kate, my darling, you must tell me what to do; for I cannot trust to my own judgment."

She held out her hand to him, in mute acceptance of the task he entrusted to her, and waited for him to begin his story.

"Long ago," he commenced, "before I knew you, or dreamed that anyone so sweet existed, I was engaged to some else."

"Oh!" Kate withdrew her hand an infinitesimal bit. A woman cannot help resenting the idea of any other having possessed her lover's heart, however remote and incomplete the possession may have been. But the momentary jealousy was followed by an instant pang of shame, and she drew nearer to Harry, and knelt by his side, in order to bring her face to the level of his down-bent head. "Tell me about her," she said gently.

"There isn't much to tell, in one way. She was very pretty; that's all."

Again a pang shot through the girl. A needless pang; for the least formidable rival to the present, among all a man's past loves, is she who has chained him only by her beauty, whom he describes as "very pretty; that's all."

"It was before my uncle married that I fell in love with her," Harry went on. "She accepted me, and I thought all was right; but after it appeared that I wasn't to be my uncle's heir she broke off the engagement. She said her mother insisted on it. I don't know; Mrs. Ellis seemed to me to be a quiet old lady who did what her daughter bade her; but Evelyn always assured me that I didn't know her real nature, and that no one could stand against her will. At any rate, the matter seemed hopeless, as she would not marry me on a moderate income, and I had no prospect of a large one. But I said to her that if she ever reconsidered her decision, and made up her mind to accept me, she was to let me know, and I would go to her at once. You see, dearest, I had never been really in love then, and so I over-rated my feeling for her."

"Yes, yes,—I understand; but go on."

"Well, she has reconsidered the matter, and she has let me know."

"Let you know—now, when your uncle and his child are dead, and you are rich once more. Oh, Harry! you can't want any help to decide a matter like that! The mercenary, horrid,—oh! I can't find words to describe her!"

"But you wrong her, Kate. It was before my uncle's death, before the baby's death, that she wrote to me. That's what makes the trouble."

"And you only tell me of it now! Why did you not think of the matter, and decide what your duty was before—before—"

"I got the letter only a few minutes ago. This is how it was. It came on the morning of the day when I met with my accident—the day I first saw you! Mitchell was in a hurry to catch the train, so I thrust it unopened into my pocket, meaning to read it in the dog-cart or somewhere, and then—well, then I forgot all about it. It was in the pocket of the coat which you cut off, and which I afterwards gave to Peter. He discovered it only two days ago, and gave it to Willis to hand to me. She did so a quarter-of-an-hour ago. Here it is. Read it, and then, Kate, my darling, tell me what I ought to do, for I cannot decide for myself."

Kate took the letter and read it through slowly and carefully, trying to judge the words impartially, and to overcome the instinctive dislike she felt to the flowing, neat, elaborately feminine calligraphy. Had she been shown the letter by a stranger, as a mere specimen of hand-writing she would have pronounced the writer to be worldly, narrow-minded, and selfish; but she put aside all such subtle, irrational verdicts, and strove to do justice to Miss Ellis's meaning.

"She seems to take it for granted that you care for her as much as ever," she remarked at last.

"Yes—unfortunately."

"You are quite sure you gave her to understand that you would come to her again if ever she called you?"

"Quite sure."

"She never said, or implied, that she did not care for you?"

"Never. It was her mother who insisted on our parting,—at least so she said; though I thought she might have defied her mother."

"Oh no, Harry. I know I would never marry against papa's wishes; and perhaps—no doubt—she felt the same."

It is needless to say that in Miss Mountstephen's inmost soul she did not believe that Evelyn Ellis had "felt the same," but she was determined to give her rival the benefit of every doubt.

"Then, Katie, what ought I to do?"

Kate was silent for a little. At last she cried out impulsively: "Oh, I wish you had asked that of any other woman in the world than me!"

"But you are the only woman I know in the world who would be sure to advise me truly and impartially."

She was silent again. If he had only taken this trouble—this one only—to some other adviser, or if the question had lain less near her own heart! Had any other than Harry Barnett brought this question before her, had not her own happiness been involved in the decision, she would have said: "Be true to the woman you love. Tell your old sweetheart that your feelings are changed, that you cannot care for her as a husband should. You will wrong her more by marrying her without love than by breaking with her now." But she was afraid to say this to him, lest it might be not justice but selfishness that spoke; lest she should be sacrificing her rival to herself. Better by far, she thought, to sacrifice herself to her rival; for Kate Mountstephen, like all good women, believed devoutly that though to take the path you do like may be right, to take the one you don't like must be so.

She spoke at last, slowly and painfully, as if the words were forced from her. "I think, since she has cared for you all the time, and has at last won her mother's consent, not guessing that the obstacles that separated you were to be cleared away, and since you have left her to believe that you still love her—I think you have no alternative but to marry her."

(*To be continued.*)

